# Novel mutation in exon11 of *PRKCG* (SCA14): A case report

**DOI:** 10.3389/fgene.2023.1129988

**Published:** 2023-03-08

**Authors:** Rong Sun, Xiang Tang, Xueqin Cao, Xinyu Shao, Hong Sun

**Affiliations:** ^1^ Department of Endocrinology and Metabolism, Dushu Lake Hospital Affiliated to Soochow University, Medical Center of Soochow University, Suzhou, Jiangsu, China; ^2^ Department of Neurology, The First Affiliated Hospital of Soochow University, Suzhou, Jiangsu, China

**Keywords:** spinocerebellar ataxia type 14, PRKCG gene mutation, case report, exon11, dystaxia

## Abstract

**Introduction:** *PRKCG* mutations have been implicated in the pathogenesis of spinocerebellar ataxia type 14 (SCA14), which is a rare autosomal dominant disease marked by cerebellar degeneration, dysarthria, and nystagmus. Until now, there has never been a report of patients with mutations of c.1232G>C worldwide.

**Case description:** We report a case of a 30-year-old Chinese man with episodic dystaxia, speech disorder, and cognitive impairment; however, his father exclusively exhibited a speech disorder regardless of the same mutation. Whole-exome sequencing revealed a heterozygous c.1232G>C (p.G411A) variant of *PRKCG*.

**Conclusion:** This case presents an extended genotype and phenotype of SCA14, and emphasizes the importance of gene sequencing in patients with spinocerebellar ataxia.

## Introduction

Spinocerebellar ataxia type 14 (SCA14) [OMIM: 605361] (Yamashita et al., 2000; Brkanac et al., 2002) (Rossi et al., 2014) is an autosomal dominant disorder characterized by progressive cerebellar degeneration, dysarthria, and nystagmus. Symptoms, such as axial myoclonus (Yamashita et al., 2000), cognitive impairment (Wedding et al., 2013; Bolton and Lacy, 2019), tremors (Koht et al., 2012), and impaired sensibility (Klebe et al., 2005; Koht et al., 2012), may also be observed. Furthermore, Parkinson’s disease (Sailer et al., 2012; Chen et al., 2022), which is characterized by muscle rigidity and tremors, has also been reported in some family pedigrees. Patients with SCA14 may exhibit further ataxic conditions such as dysphagia (Ueda et al., 2013). The incidence of SCA14 is from 1% to 4% in all autosomal genetic disorders (Chelban et al., 2018), and it was first reported in a Japanese family in 2000 (Yamashita et al., 2000). It was also reported in a 4th generation American family of English and Dutch origin who displayed pure cerebellar ataxia (Brkanac et al., 2002). SCA14 has also been reported in various countries such as Australia (Kang et al., 2019), Norway (Koht et al., 2012), Germany (Ganos et al., 2014), Japan (Ueda et al., 2013), and China (Chen et al., 2022). There is an ambiguous correlation between clinical manifestations and ethnicity, while the age of onset occurs between childhood and 60 years old. Diagnosis is mainly based on clinical manifestations, physical examination, and laboratory tests, and genetic testing is required to confirm the diagnosis. SCA14 is caused by *PRKCG* variants encoding protein kinase C *γ* (PKC*γ*) (Yabe et al., 2003). Although missense and deletion mutations have been found in *PRKCG* (Chelban et al., 2018), the specific molecular mechanism underlying pathogenesis remains poorly understood (Shimobayashi and Kapfhammer, 2021). Here, we identified a c.1232G>C mutation in *PRKCG* in a Chinese family to extend the genotype and phenotype of SCA14. Our results emphasize the importance of detecting *PRKCG* mutations in patients with episodic ataxia.

## Case description

### Clinical characteristics

**FIGURE 1:**
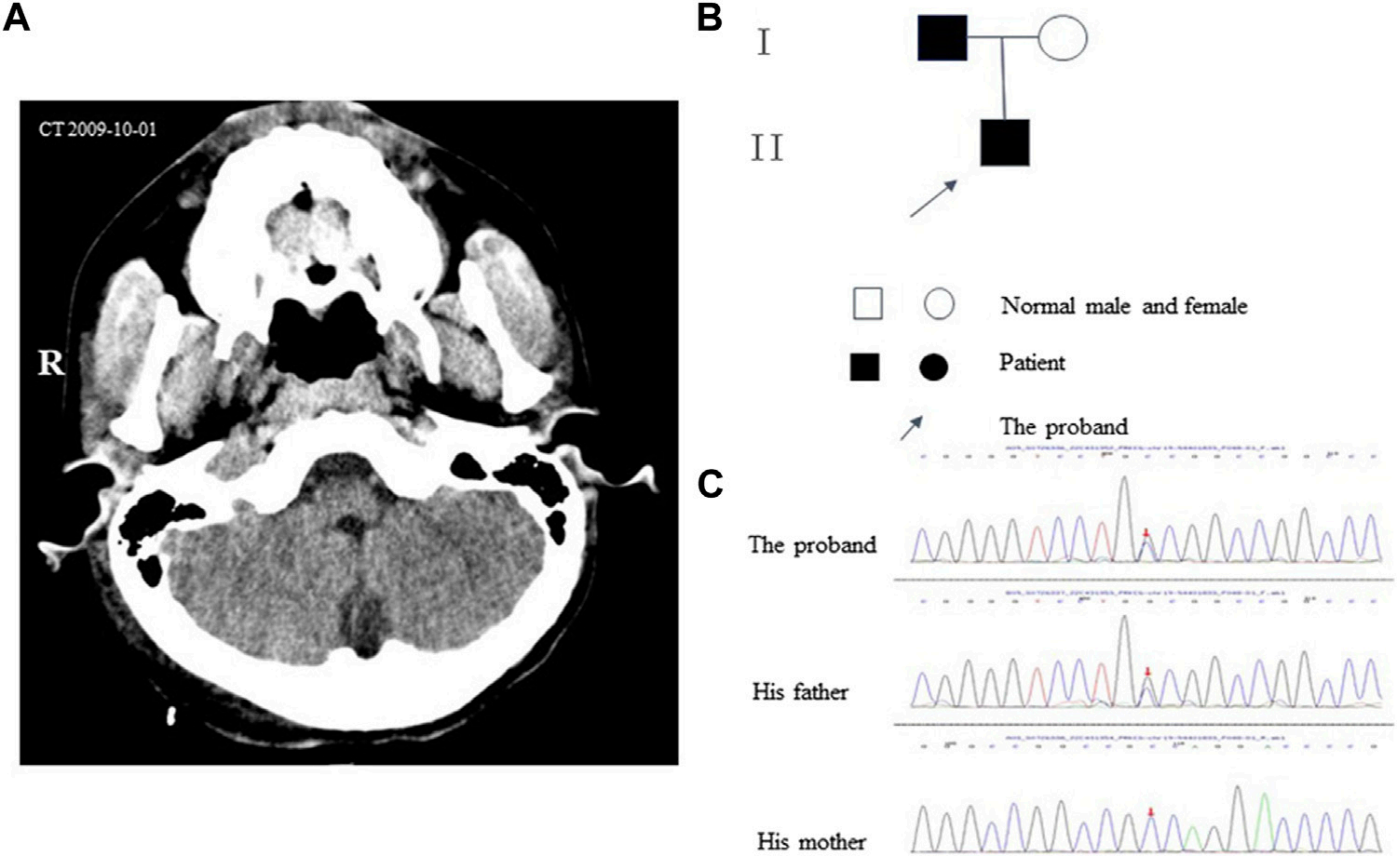
**|** **(A)** Axial CT images showing the cerebellum. **(B)** Pedigrees of the family. **(C)** The result of gene sequencing of the *PRKCG* gene. The arrow indicates the c.1232 (exon11)G>C.

**FIGURE 2:**
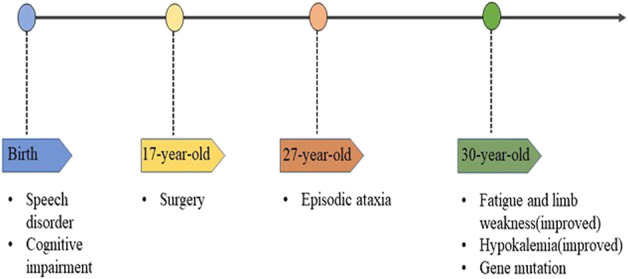
The timeline of the proband.

The proband (aged 30) suffered from episodic ataxia for 3 years and was hospitalized for hypokalemia in the Endocrinology Department, Dushu Lake Hospital Affiliated with Soochow University on 6 July 2022. The patient was experiencing fatigue and limb weakness after heavy sweating in hot weather. He had experienced a speech disorder and cognitive impairment since birth and his speech was characterized as slow and slurred. A neurological examination revealed that his gait was ataxic and his tandem gait was impaired. The tendon reflexes were normal, and Hoffman and Babinski’s signs were negative. No axial myoclonus or tremors had been observed during the past 30 years. There was a requirement for assistance while transferring (activities of daily living score: 95). After admission, a complete examination revealed that the Renin–angiotensin–aldosterone system was normal. The potassium level in the 24 h urine sample was 64.58 mmoL/L (normal range: 25–125 mmoL/L), and the calcium level was 0.59 mmoL/L (normal range: 2.5–7.5 mmoL/L). Because the patient suffered from headaches, a right-sided parietooccipital tumorectomy was performed in 2009, and the postoperative pathology suggested an arteriovenous malformation. The postoperative magnetic resonance imaging (MRI) and computerized tomography (CT) scans (Figure 1A) suggested postoperative changes; however, cerebellar atrophy changes were not observed. The proband had no siblings, and his grandparents and aunt did not exhibit any clinical manifestations, but his father had a speech disorder. The pedigree of the proband is shown in Figure 1B. During hospitalization, the patient’s hypokalemia was corrected, and the weakness in his limb was improved after administering potassium supplementation; however, episodic ataxia remained without remission. The patient refused further follow-up and treatment, which limited the process of collecting more clinical data. The timeline with relevant data is shown in Figure 2.

### Genetic results

**FIGURE 3:**
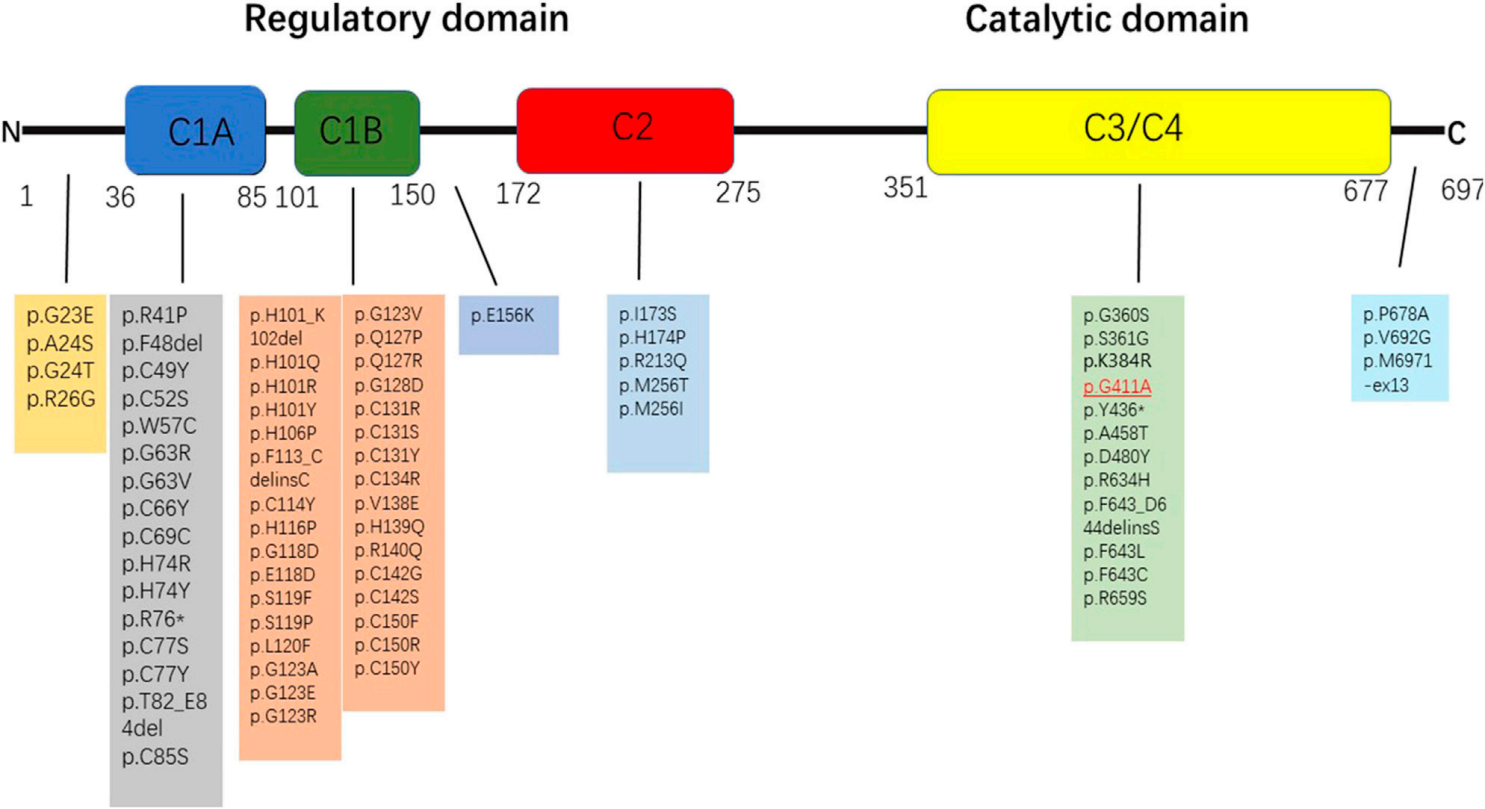
Variants associated with ataxia that are described in the protein kinase C γ coded by *PRKCG*. The pathogenic or likely pathogenic variants identified in this study are highlighted in red.

Peripheral blood in EDTA was collected for whole genomic extraction. The proband and his parents underwent genetic testing, but his grandparents and aunt were not considered for genetic testing because they were asymptomatic. The mutation was verified in samples procured from family members by Sanger sequencing. The verification revealed the presence of a heterozygous mutation in *PRKCG* c.1232G>C (p.G411A) (Figures 1C, 3). The proband’s father had the same heterozygous mutation. According to the guidelines developed by the American College of Medical Genetics and Genomics (ACMG) for the classification of pathogenic or likely pathogenic variants, the *PRKCG* mutation was classified under “uncertain significance” on the basis of the evidence framework of PM2 and BP4. The *PRKCG* sequence (NM_002739) was obtained from the National Center for Biotechnology Information (NCBI) (https://www.ncbi.nlm.nih.gov/). The three-dimensional model of PRKCG protein was obtained from the AlphaFold Protein Structure Database (https://alphafold.ebi.ac.uk/). The three-dimensional models of the wild-type and p.G411A (c.1232G>C) mutant proteins were generated by PyMOL 2.5, a protein three-dimensional structure visualization software (Jumper et al., 2021).

## Discussion and conclusion

The clinical manifestations of SCAs, which are a unique type of cerebellar degenerative disease, range from typical ataxia disturbance to abnormal ocular movements. Many patients exhibit cerebellar ataxia, but this may not be an early symptom. In a previous case, dystonia was reported as the only symptom present in the early stages (De Michele et al., 2022). In this case, the proband had a speech disorder and cognitive impairment since birth and suffered from episodic ataxia over the last 3 years. Clinical manifestations and imaging results revealed that there were no traces of postoperative complications such as hydrocephalus and herniation. Furthermore, we consulted a neurosurgeon and a neurologist at Dushu Lake Hospital Affiliated with Soochow University. Furthermore, it has been 10 years since the right-sided parietooccipital tumorectomy was performed, and the cerebellar ataxia of the patient was not considered to be caused by a craniotomy. Considering the episodic ataxia, mild cognitive impairment, and speech disorder, it was recommended that the patient undergo genetic testing. Whole genomic extraction showed mutations in *PRKCG* with no mutations in *KCNA1* or *CACNA1A*, which are commonly observed in episodic ataxia. Based on the genotype and phenotype, it was concluded that repeated falls were caused by ataxia rather than hypokalemia. Moreover, during previous episodic ataxia, the patient did not undergo strenuous exercise or exhibit a loss of appetite, vomiting, diarrhea, and other causes of low potassium levels. Additionally, no relationship between SCA14 and hypokalemia was reported. The absence of ataxia and cognitive dysfunction in the patient’s father, who had the same genotype, may be explained by the decreased penetrance of the disorder (Yabe et al., 2003).

SCAs are a group of progressive neurogenetic disorders caused by gene mutations. SCA14 is a rare autosomal dominant inherited disease involving *PRKCG* mutation. *PRKCG* encodes protein kinase C *γ* (PKC*γ*), which is a member of the serine/threonine enzyme family, plays a vital role in cell growth and signal transduction, and is highly expressed in Purkinje cells (Winkler et al., 2020; Shimobayashi and Kapfhammer, 2021). The activity of PKC*γ* also plays a major role in dendritic development and synaptic maturation of cerebellar Purkinje cells (Winkler et al., 2020). In vitro studies and studies on transgenic mice (Winkler et al., 2020) support the functional gain hypothesis, which indicates that increased PKC*γ* activity leads to dendritic dysplasia, neuronal death, and aggregation effects. The effect of PKC*γ* is related to the membrane residence duration (Wong et al., 2018). However, a study has shown that PKC*γ* exerts toxic effects by inducing endoplasmic reticular stress (Seki et al., 2007). Shimobayashi (Shimobayashi and Kapfhammer, 2017) reported that SCA14 depends on the activity of PKC*γ*, and mutations in different domains occur due to different pathways.

**FIGURE 4:**
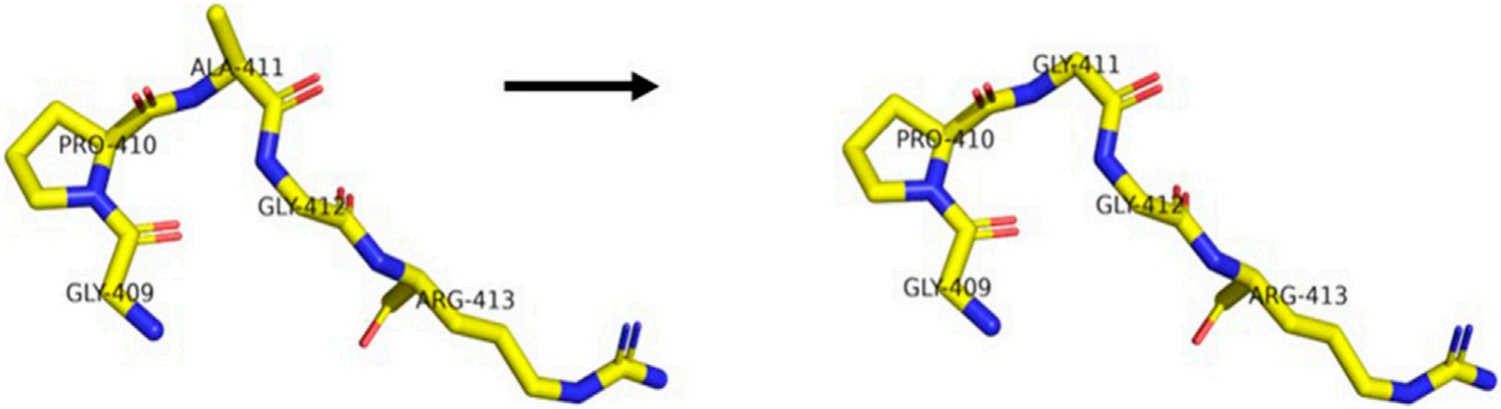
Difference between the wide-type and mutant protein (p.G411A) of *PRKCG* (regional model). The left model is the mutant, and the right model is the wide-type.

Until now, many mutation sites have been reported (Chen et al., 2022; De Michele et al., 2022; Tada et al., 2022) (Schmitz-Hübsch et al., 2021) (Figure 4) in *PRKCG*. However, we pioneered the determination of a novel heterozygous mutation in *PRKCG* c.1232G>C (p.G411A). *PRKCG* consists of two domains, namely, the regulatory domain and the catalytic domain. The regulatory domain consists of the C1 and C2 regions. The majority of the mutations discovered in the C1 region are missense variants (De Michele et al., 2022). Variants in the catalytic domain are rare, and the clinical phenotype is much more complex (Chelban et al., 2018) because of the inhibition of calcium ion inflow (Adachi et al., 2008), which in turn prevents dendritic processes, as reported in this case. The mutation gene is located in exon11, which is in the catalytic domain. The patient had a speech disorder and cognitive impairment since birth. The disease progressed and ataxia initially occurred 3 years ago. However, his father had the same mutation that only manifested as a speech disorder. Transgenic mice (Ji et al., 2014) with PKCγ mutations located in the catalytic domain (S361G) have shown pathological changes and motility defects typical of cerebellar ataxia, which is consistent with this case. However, the mutation (p.G411A) reported here requires further functional verification.

Many patients diagnosed with SCA14 display progressive cerebellar syndrome, which is rarely associated with severe disabilities (Koht et al., 2012). Dysarthria, abnormal ocular movements, and dysmetria are the most common clinical manifestations, and cognitive impairment is rare and is usually mild (De Michele et al., 2022) in these patients. To summarize the clinical features of SCA14 reported thus far, they can be divided into two categories as follows: the classic type and the atypical type. The characteristics of the classic type are as follows: 1) cerebellar ataxia, most patients present with slowly progressive ataxia; 2) dysarthria or a speech disorder; 3) ocular symptoms, abnormal ocular movements, and dysmetria; and 4) cerebellar atrophy in the MRI. The characteristics of the atypical type include cognitive impairment, depression, and epilepsy. All affected subjects that were considered in this article had a speech disorder, and the proband exhibited progressive ataxia and mild cognitive dysfunction, which is consistent with the clinical manifestations of a previously reported case of SCA14 (Wedding et al., 2013). The proband had no cerebellar atrophy, as revealed by a CT scan on 1 October 2009, and he refused to undergo more MRI scans due to personal reasons. However, if the patient exhibits the development of cerebellar atrophy in the future, a further examination will be required. To summarize, SCA14 should be considered when patients exhibit the following features: slowly progressive cerebellar ataxia, dysarthria or a speech disorder, abnormal ocular movement, extrapyramidal systemic syndrome, and mild to severe atrophy of the cerebellum revealed by brain magnetic resonance. Presently, a diagnosis for the same relies on gene detection. The majority of patients with SCA14 have a long history of the disease; thus, ordinary life is unaffected. However, many patients may die from dysphagia or falls. Currently, there is no targeted therapy for SCA14. Supportive treatment includes heteropathy, such as rehabilitation treatment, and antisecosis to reduce the risk of asphyxia.

We reported on this family of Chinese origin with episodic ataxia, speech disorders, and cognitive impairment with an extended genetic profile and clinical features of SCA14, and emphasize the importance of gene sequencing in patients with episodic ataxia regardless of the absence of classic dysmetria and nystagmus and also independent of familial history.

## Data Availability

The datasets presented in this study can be found in online repositories. The names of the repository/repositories and accession number(s) can be found below: MedGen UID: 343106; Concept ID: C1854369; Accession SCV003798486.
